# Multiple Autoregulation of Nodulation (AON) Signals Identified through Split Root Analysis of *Medicago truncatula sunn* and *rdn1* Mutants

**DOI:** 10.3390/plants4020209

**Published:** 2015-04-27

**Authors:** Tessema Kassaw, William Bridges, Julia Frugoli

**Affiliations:** 1 Department of Genetics & Biochemistry, Clemson University, Clemson, SC 29634, USA; E-Mail: Tessema.Kassaw@colostate.edu; 2 Department of Mathematical Sciences, Clemson University, Clemson, SC 29634, USA; E-Mail: wbrdgs@g.clemson.edu; 3 Department of Genetics & Biochemistry, Clemson University, Clemson, SC 29634, USA

**Keywords:** autoregulation of nodulation, *Medicago truncatula*, split-root analysis, nitrogen, *RDN1*, *SUNN*

## Abstract

Nodulation is energetically costly to the host: legumes balance the nitrogen demand with the energy expense by limiting the number of nodules through long-distance signaling. A split root system was used to investigate systemic autoregulation of nodulation (AON) in *Medicago truncatula* and the role of the AON genes *RDN1* and *SUNN* in the regulatory circuit. Developing nodule primordia did not trigger AON in plants carrying mutations in *RDN1* and *SUNN* genes, while wild type plants had fully induced AON within three days. However, despite lacking an early suppression response, AON mutants suppressed nodulation when roots were inoculated 10 days or more apart, correlated with the maturation of nitrogen fixing nodules. In addition to correlation between nitrogen fixation and suppression of nodulation, suppression by extreme nutrient stress was also observed in all genotypes and may be a component of the observed response due to the conditions of the assay. These results suggest there is more than one systemic regulatory circuit controlling nodulation in *M. truncatula*. While both signals are present in wild type plants, the second signal can only be observed in plants lacking the early repression (AON mutants). *RDN1* and *SUNN* are not essential for response to the later signal.

## 1. Introduction

When legumes set up a symbiosis with bacteria in the genus *Rhizobia*, the plants develop a new organ on the roots (a nodule) to house the bacteria. After entering the plant through complex species-specific recognition and growth events, the bacteria reach the cortical cells of the root, differentiate into bacteriods and fix nitrogen in the low oxygen environment provided by the plant, sharing nitrogen in exchange for carbon skeletons from photosynthesis (for review see [1]). The nitrogenase enzyme used by the bacteria to fix N_2_ into useable ammonia is irreversibly inhibited by oxygen and requires the plant to provide this special environment. Although the symbiosis provides nitrogen to the plant, allowing legumes to thrive under conditions other plants cannot, legumes only enter into the symbiosis under nitrate limitation. Suppression of nodulation in the presence of nitrate is thought to occur because the formation and maintenance of the nodule environment is energetically costly to the plant; the biological cost to the plant of nodule establishment, nitrogen fixation and transport is estimated at 12–17 grams of carbon per gram of nitrogen [2]. Likewise, plants that have already entered into the symbiosis will suppress the development of future nodules, even before the original nodules become fully established and provide ammonia [3], suggesting that there are several molecular switches controlling nodule regulation or that the regulation requires coordination of a number of signals.

The suppression of future nodule development is termed autoregulation of nodulation (AON) and it involves long distance signaling from root to shoot and back again; long distance signaling was initially demonstrated in split root inoculation experiments by first inoculating one side of the split root and observing systematic suppression of further nodulation in the other side of the split root [3]. AON is activated early upon Nod factor perception; the elongation zone of the root with emerging root hairs is vulnerable to rhizobial infection and is the portion of the root affected by the autoregulation process [4]. The timing of full induction of AON varies from species to species and is influenced by growth conditions. AON induction in *G. max* has been observed within four days [3] and in *L. japonicus* within five days [5], but reports of shorter times of two days in *Trifolium subterraneum* (clover) [6] and 30 h in *Vicia sativa* [7] have also been published.

Both shoot and root controlled AON defective mutants have been reported in *Lotus japonicus*, *Pisum sativum*, *Glycine max* and *Medicago truncatula*. The first shoot controlled AON gene cloned was *HAR1/SYM29/NARK/SUNN*, encoding a leucine-rich repeat receptor protein kinase like CLAVATA1 (CLV1) of *Arabidopsis thaliana* [8,9,10,11]. The protein kinase has an extracellular domain of leucine-rich repeats, a membrane spanning domain and an intracellular protein kinase domain. Mutations in *SUNN* (*super numerary nodules*) result in a five- to tenfold increase in nodule number [11]. Because the mutations exert their effect from the shoot, they are believed to be the receptors for the root-derived CLE signal [12] and/or triggers for the biosynthesis and release of the shoot derived inhibitory signal [13]. Among the genes known to exert their effect from the root is the *RDN1/NOD3* gene (*r*oot-*d*etermined *n*odulation) in *M. truncatula* and pea [14]. The *RDN* gene family in *M. truncatula* is orthologous to the *HPAT* gene family in *Arabidopsis*, which has been shown to add arabinose residues to the hydroxyprolines of some CLE peptides [15]. CLE peptides are attractive as the root derived signal, and in *M. truncatula* the expression of two CLE peptides are correlated with nodule development. The *MtCLE12* and *MtCLE13* genes are induced in the root upon nodulation, are expressed in nodule meristems and overexpression reduces nodulation in a SUNN dependent manner [16,17,18,19]. RDN1, therefore, presumably, affects the release of the systemic signal received by SUNN. Plants homozygous for either *rdn1-2* or *sunn-4* alleles result in excessive nodulation, with the *sunn-4* mutation giving the most severe effect [14,20]. 

In wild type plants, successful nodule formation and subsequent nitrogen fixation occur only under nitrogen limiting conditions. In the presence of high concentrations of biologically available nitrogen, plants cease nitrogen fixation and nodule formation is suppressed [21]. In contrast, soybean mutants with defects in AON are reported to be nitrate tolerant, nodulating abundantly even in the presence of nitrate [22,23]. In *M. truncatula*, the plants carrying mutations in *SUNN* or *RDN1* nodulate in the presence of nitrate, but at reduced levels compared to when nitrate is lacking [14]. This suggests that an autoregulation signal and a nitrate signal both act to inhibit nodule formation. Fixed nitrogen is transported in the xylem of nodulators largely as glutamine, asparagine or uriede [24]. The large pool of asparagine that is present in nodules may buffer the transport of nitrogen and thus act to regulate nitrogen fixation via a feedback mechanism [24]. In addition, inhibition of nitrogen fixation can affect the partitioning of carbon and other metabolites from the shoot. Singleton and van Kessel showed that the flux of photosynthate to nitrogen fixing nodules and their associated roots is greater than that to non-nitrogen fixing sections of the root [25], suggesting that nitrogen assimilation, either from N_2_ fixation or inorganic sources, has a localized effect on both nodule and root development. Split root systems have also demonstrated that the lack of nitrogen acquisition by a half root system nodulated with non-fixing *rhizobia* triggers a compensation response, enabling the other half root system nodulated with nitrogen fixing partners to compensate for the local nitrogen limitation of the first root [26], which suggests a systemic component as well. 

In order to determine if the excess nodule number phenotypes of the *rdn1* and *sunn* mutants involve changes in AON timing, we investigated AON in wild type plants and plants carrying mutant alleles of *RDN1* or *SUNN*. We report an early AON signal evident at three days in wild type but not mutant plants and a second AON signal ten days later observed in the AON mutants. Our findings using measurements of nitrogen fixation, response to supplied nitrogen and inoculation with bacteria unable to fix nitrogen support the assertion that the second AON signal is not associated with early nodulation events but rather with the establishment of nitrogen fixing nodules and although this signal is presumably masked by the CLE12/13-SUNN signaling in wild type plants, it is unaffected and detectable in the *sunn* and *rdn1* AON mutants. Additionally, both wild type and AON mutant plants suppress nodulation under severe nutrient stress, suggesting that the nitrate and stress signals are not affected in AON regulatory mutants.

## 2. Results and Discussion

### 2.1. AON Mutants Lack an Early Systemic Response to Previous Nodulation Events, but Suppression Returns after 10–15 Days

**Figure 1:**
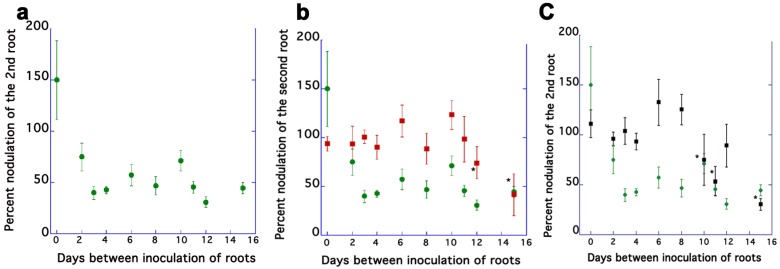
Persistence of the systemic suppression signal in mutant and wild type plants. (**a**) Wild type plants have consistently fewer nodules on the second root when the second root is inoculated 2–15 days after the first. Percent nodulation was calculated by dividing the mean nodule number of the second root by the mean nodule number of the first root. Values for time points 2–15 are statistically significant from time 0. (**b**) Wild type values (green dots) compared to the *sunn-4* allele values (red boxes). Data points marked with * are not statistically different between wild type and mutant (**c**) Wild type values (green dots) compared to the *rdn1-2* allele values (black boxes). Data points marked with * are not statistically different between wild type and mutant. Percent nodulation was calculated by dividing the mean nodule number of the second root by the mean nodule number of the first root. Statistical comparisons among means are based on factorial ANOVA followed by pairwise mean comparisons. Data are shown as means ± SE. (*n* = 6 to 47 plants for each time point per genotype).

As noted in the introduction, the timing of AON has been reported to vary between species. Although we were able to observe autoregulation at four days post inoculation in previous work [27], we refined the experiment to determine the duration of the systemic autoregulatory signal timing in *M. truncatula* using the split-root system in [27]. Plants with two spatially separated equal roots, a tester (Root A) and a responder (Root B) were inoculated with *S. medicae* simultaneously or from two to 15 days apart. We compared the nodulation of Root B to the nodulation of Root A 21 days after the inoculation of root B, reporting the results as a percentage of the total nodules on Root A to account for the higher nodule numbers of AON mutants. When inoculated simultaneously, the responder roots and tester roots of wild type had no significant difference in nodule number (Figure 1a). The value of 150 percent for the mean at Time 0 results from the need to randomly designate one root as Root A when calculating the mean percentage of a simultaneous inoculation and wild type roots inoculated at the same time varied as much as 5–15 nodules/root. Percent nodulation on the responder roots decreased in wild type when the roots were inoculated two days apart, and as the interval between inoculations increased from three days to 15 days, the responder root consistently developed approximately half of the nodules of the first root, a statistically significant difference. Distribution of the nodules on Root A and Root B were similar, consisting of some nodules in the lower parts of the roots and some in developing laterals near the surface. This indicated that AON occurs between two and three days in *M. truncatula* and the degree of suppression remains constant through at least 15 days. 

Unlike reports in soybean [28], we did not observe significant suppression under our conditions until day three and nodulation of the root portion receiving the delayed inoculation (Root B) was never completely suppressed by prior inoculation of the other root (Root A); even at a 15-day interval between inoculation of the roots suppression hovered around 50%. The three-day interval for full induction of AON in *M. truncatula* is shorter than previous reports in soybean and *L. japonicus* [3,5], but longer than that those reported for clover and *Vicia sativa* [6,7]. This variation could be due to differences in the split root systems used to determine the timing (see discussion in [27]), a difference between species in the speed of translocation of the signal, morphological and anatomical differences in the species, or the different rhizobial partners for each legume species.

The same analysis was performed using hypernodulation mutants carrying a null allele of the *SUNN* gene, *sunn-4* [11], which contains a stop codon very close to the initial signal sequence or a null allele of the *RDN1* gene, *rdn1-2* [14], which contains an insertion near a splicing site that reduces RNA levels over 400 percent. As expected for mutants isolated for their defects in AON, no significant difference in nodulation percentage between Root A and Root B was observed for inoculations separated by zero to eight days (Figure 1b,c). However, at ten days between inoculations in *rdn1* mutant plants and 12 days in *sunn-4* mutants, we observed suppression of nodulation to wild type suppression percentage levels in Root B at all but one time point. At data points separated 10 days or more between inoculations, the nodules on Root A were pink (a likely indication of active nitrogen fixation because of leghemoglobin synthesis) and thus the suppression coincides with the transition from white nodules to pink nodules, which occurs between eight and 10 days in this system. Nitrogen fixation is a possible explanation for the source of the later regulatory signal. Nitrogen fixation creates a strong localized sink for photosynthate that has effects on both nodulation and root development in uninoculated portion of the roots [25] and application of high inorganic nitrogen affects nodulation [29].

### 2.2. AON Mutants and Wild Type Fix Equivalent Amounts of Nitrogen at Time Points when Systemic Suppression Is Observed

**Figure 2:**
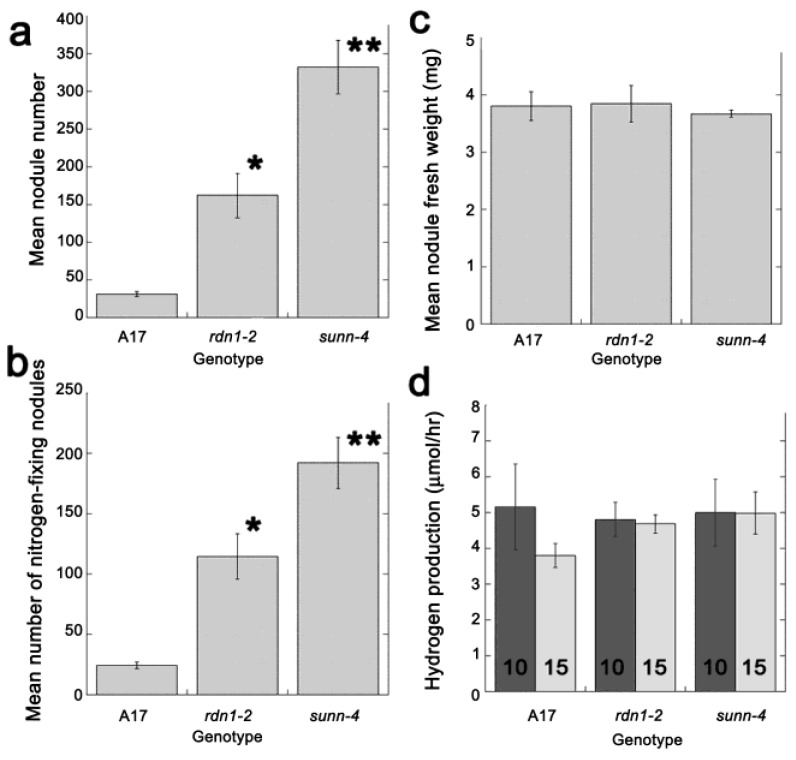
AON mutants and wild type plants fix equivalent amounts of nitrogen. Roots of A17, *rdn1-2* and *sunn-4* plants were evaluated 15 days after inoculation with *S. medicae*. Means ±se are per genotype (**a**) Mean nodule number (**b**) Mean number of nitrogen fixing (pink) nodules (**c**) Mean nodule fresh weight (ten samples of three nodules each from five to six plants per genotype). (**d**) Hydrogen production (μmol/hr) as a proxy for nitrogen fixation rate (see Experimental Section). Measurements were taken on the same plants at both 10 and 15 days after inoculation with *S. medicae* in two biological replicates. A17 (*n* = 9), *rdn1-2* (*n* = 10) and *sunn-4* (*n* = 9). Statistically significant differences from wild type based on Student’s t-test are indicated by * (*p* < 0.05) and ** (*p* < 0.01).

Since we postulated that the mutants were supressing nodulation on the responder root in response to nitrogen fixed by the tester root, to pursue assimilated nitrogen as the signal for the systemic suppression, we compared measures of nitrogen fixation capacity between AON mutants (*rdn1-2* and *sunn-4*) and wild type plants at the time point in question. First, we compared the total number of nodules (Figure 2a), the number of nitrogen fixing nodules (Figure 2b), and the mean nodule fresh weight (Figure 2c) between mutant plants and wild type plants. Both the total and nitrogen fixing nodule numbers were significantly higher in the AON mutants than in the wild type plants, but there was no difference in mean nodule fresh weight, in agreement with greenhouse work on the orthologous mutants in pea [30]. We then measured the rate of nitrogen fixation at 10 and 15 days post inoculation with *S. medicae* in wild type plants and the AON mutants, since despite having more nitrogen fixing nodules, the level of pink color varied between nodules on mutant and wild type plants. However, the level of nitrogen fixation in plants 10 and 15 days after inoculation was the same as wild type in all mutants (Figure 2d), suggesting that *sunn4* and *rdn1-2* mutants compensate for increased nodule number with reduced nitrogen fixation activity per nodule. In support of this, Jeudy *et al.* [31] showed that the systemic regulation of nitrogen fixation activity by the plant is *SUNN* independent; excess capacity in the *sunn-2* mutant allowed it to up regulate nitrogen fixation activity in response to localized nitrogen starvation in a split root system. 

Thus the “nitrogen fixation signal” would be the same strength in wild type and AON mutants. Since both wild type and AON mutant plants reach the same level of nitrogen fixation at 10 days post inoculation, it is possible the systemic repression observed is a response to nitrate acquisition. We note it would not be possible to detect this second suppression in wild type plants in the split root system because wild type plants have already suppressed nodulation on the second root due to CLE12/13-SUNN signaling from developing nodules at the time this second signal is sent.

### 2.3. Nitrate Reduces Nodulation in Both AON Mutants and Wild Type Plants in this System

**Figure 3:**
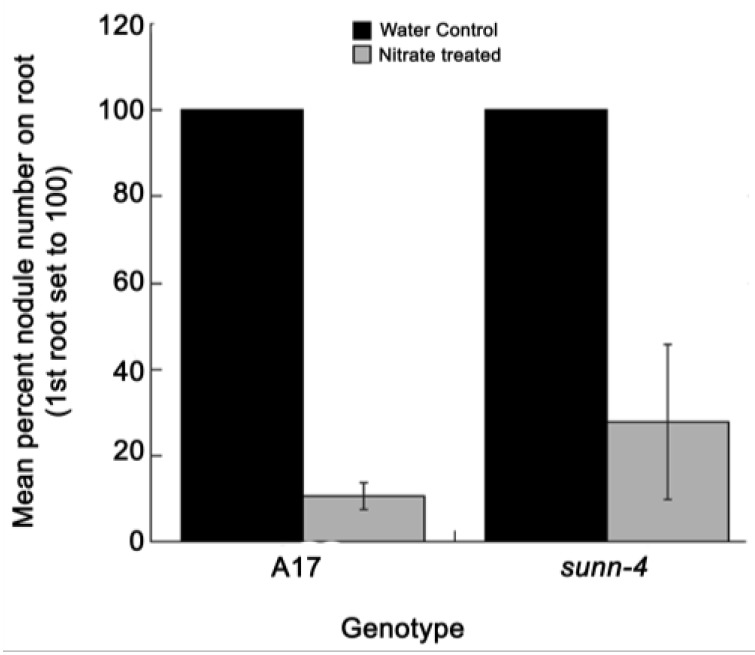
Effect of ammonium nitrate *versus* water treatment of first root on nodulation of second root in a split root system. Split root plants were grown as in [27] and the first root fed with either water or 10 mM NH_4_NO_3_ for four days before inoculating the second root. Nodules were counted on the second root of A17 (wild type) plants (*n* = 12) and *sunn-4* plants (*n* = 7) 20 days after inoculation and expressed as percentage of the water controls. Bars indicate standard error of the percentage means calculated by Taylor series expansion.

In previous work, we demonstrated that in an aeroponic system, supplying *M. truncatula* with nitrate reduced nodule number in both wild type and the AON mutants *sunn* and *rdn1* [14]. To confirm the results applied to our perlite split root system, we tested the systemic nature of the effect in one of the mutants (*sunn-4*) and wild type. Roots were starved for four days (water only) and then the first root was supplied with nitrate for four days in half of the plants while the control plants’ first roots continue to receive only water. Four days later, the second root is inoculated with *S. medicae* and nodules on all roots are counted 20 days later. When nodules on the second root are reported as a percent nodulation of the first root to account for the higher nodule number of an AON mutant, both the wild type and *sunn-4* mutants reduce nodule number in the second root to 10%–30% of the water control (Figure 3) and there is no statistical difference in the degree of nodule number reduction. This confirms that the effect is systemic and that defects in AON do not affect the plant’s ability to respond to available nitrate. AON mutants are commonly reported as having nitrogen resistant nodulation, in that they nodulate in the presence of nitrate while wild type plants make few to no nodules, but if viewed as a percentage reduction in nodule numbers, our data confirm the plants respond to available nitrate to the same degree as wild type, they just start from a higher number of nodulation events. Thus if nitrate suppression of nodulation is seen as a proportional rather than a binary response, there is no defect in this response in *M. truncatula* AON mutants.

### 2.4. Inoculation with Fix^−^ or Nod^+^/Fix^−^ *Rhizobia* Did Not Elicit the AON Response in all Plants

To further support nitrate as the signal for the second suppression event, we designed a split root experiment in which the first root is inoculated with Fix^−^ bacteria, which make nodules but do not fix nitrogen, or Nod^−^ bacteria which do not elicit nodule formation at all. Inoculum was switched from the natural symbiont of *M. truncatula*, *S. medicae*, to the commonly used symbiont of *M. sativa*, *Rhizobium meliloti*, to take advantage of the large number of mutant strains available in *R. meliloti*. We chose three *S. meliloti* strains: wild type *Rm*1021 [32], a Nod^+^/Fix^−^ bacterial mutant; *Rm*1312 [33] to test for nitrogen fixation response; and a Nod^−^/Fix^−^ bacterial mutant SL44 [34] to test for response to developing meristems (the CLEs). The tester root was inoculated with one of the three strains and the responder root was always inoculated with the wild type strain 20 days after the tester. This later time point was chosen to accommodate the slightly slower pace of nodule development we observed in preliminary experiments when wild type plants were inoculated with *R. meliloti versus S. medicae.*

**Figure 4:**
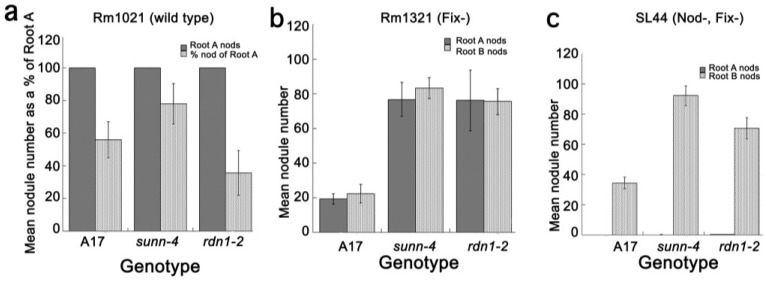
The effect of prior inoculation of the tester root with different strain of *S. meliloti* on subsequent nodulation of the responder root in A17, *sunn-4,* and *rdn1-2*. The responder root was inoculated with *Rm*1021 20 days after the tester root inoculation and nodules were counted 21 days later. (**a**) *Rm*1021 wild type on both roots, expressed as mean nodulation % of the responder root to the tester. Errors are standard error by Taylor expansion. When inoculating with different strains, data is expressed as mean nodule number ± se on the responder root to mean nodule number ± se for strains used on the tester root in (**b**) *Rm*1312 (Nod^+^/Fix^−^), and (**c**) SL44 (Nod^−^/Fix^−^). Error bars for (**b**) and (**c**) are standard error of the mean (*n* = 5 to 10 plants per genotype per experiment).

Inoculating the tester root of wild type and *rdn1* mutant plants with the wild type *Rm*1021 strain resulted in suppression of nodule number on the responder root 20 days later in *rdn1-2* mutant and wild type plants (gray bars in Figure 4a) at levels consistent with those seen with the *S. medicae* strain, indicating that the suppression shown in Figure 1 also occurred with this strain of *rhizobia* in these plants. Interestingly, *sunn-4* mutants decreased nodulation, but not to a level of statistical significance, suggesting the suppression response was muted in this genotype when inoculated with *R. meliloti*. However, inoculation of the tester root with the Nod^−^/Fix^−^ SL44 strain, which does not form nodules or nodule meristems, did not suppress nodulation on the responder root in all plants tested (Figure 4c), consistent with the postulate that without the initial signaling that leads to nodule meristems, AON does not occur in any plant. More importantly, inoculation of the tester root with the Nod^+^/Fix^−^ bacterialmutant *Rm*1312 resulted in no suppression of nodulation in the responder root of wild type or hypernodulation mutant plants (Figure 4b), suggesting that the ability of the bacteria to fix nitrogen was critical to the regulation observed in wild type plants at 20 days after inoculation, and to the late regulation phenomenon we observed in the mutants. In all plants tested, lack of nitrogen fixation in the first root abolishes regulation in the second root if the second root is inoculated after the 10–12 day time point.

### 2.5. Nutrient Stress Causes Suppression of Nodulation in Wild Type and AON Mutants

**Figure 5:**
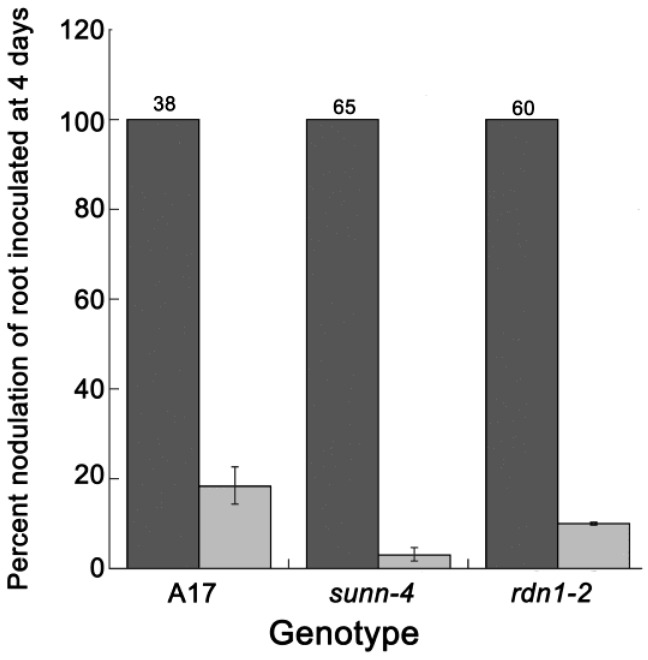
AON mutants and wild type plants suppress nodulation after long periods of water only treatment of the first root. Dark grey bars represent nodulation on the second root after four days of water treatment prior to inoculation and is set to 100% (actual mean on top of bar); light grey indicates mean percent reduction in nodulation on the second root after 11 days treatment of the first root prior to inoculation. Bars indicate standard error of the percentage means calculated by Taylor expansion. *n* = 9–16 plants per genotype per treatment.

Although we could not detect this second signal in wild type plants, we reasoned that if nitrate acquisition was the second systemic signal the AON mutants were responding to, treating the first root with water up until the time at which we observed the suppression (11 days) should abolish the suppression and the second root should hypernodulate in AON mutants and nodulate normally in wild type plants. Using wild type, *sunn-4* and *rdn1-2* plants in the split root system, the first root received only water and the second root was inoculated either after four days (the control mimicking the early conditions in Figure 1) or after 11 days. The value for the first root is set to 100 percent in Figure 5. As expected, after four days of water treatment wild type plants nodulated robustly while the AON mutants displayed significantly higher nodule numbers than wild type plants on the second root (*p* = 0.009 and *p* =0.012 for *sunn-4* and *rdn1-2,* respectively, Students *t* test). We were surprised to find that after 11 days of water, not only did wild type plants significantly reduce nodule number on the second root (*p* < 0.00001), but *sunn-4* and *rdn1-2* plants did as well, and nodule numbers on the second root of AON mutant split root plants were statistically the same as the second root of wild type plants (*p* = 0.0001). This effect does not rule out the nitrate availability signal as the source of the suppression seen in Figure 1, but it suggests that the stress of being starved for nitrogen and other nutrients also leads to a suppression of nodule number and both these suppression pathways are intact in wild type and AON mutants.

**Figure 6:**
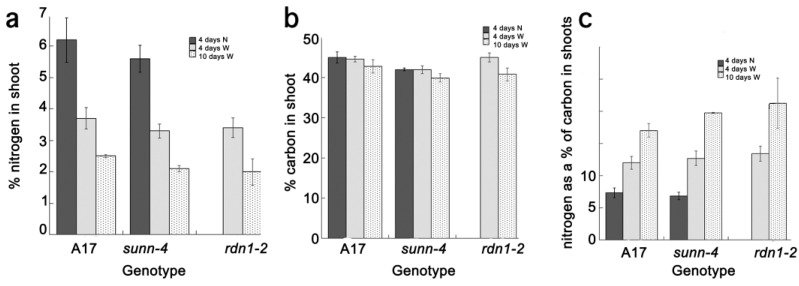
Carbon and nitrogen in shoots as a percentage of dry weight determined by ^13^C and ^15^N isotope analysis of organic solids. An equal amount of tissue was pooled from three plants for each genotype and condition at the day of inoculation, dried, and analysis performed in triplicate (**a**). Mean percent nitrogen in leaves from plants in which the first roots was watered with 10 mM NH_4_NO_3_ for four days from Figure 3 (black bars-data for *rdn1-2* not available) with water for four days from Figure 5 (grey bars) and with water for 10 days from Figure 5 (dotted bars) (**b**). Mean percent carbon from the same material and (**c**) C/N ratio of plants in (a) and (b).

To confirm that the plants were indeed stressed for nutrients, we performed C and N analysis on shoot tissue from these same plants and compared them to the plants in Figure 3 that received nitrate for four days. All tissue was harvested on the day of inoculation. After four days of treatment, as expected, plants that received nitrate had almost twice the nitrogen of plants that received only water and there was no difference between genotypes (Figure 6a). Plants which received only water on the first root for 10 days had tissue with significantly less nitrogen than the water treated plants at four days, indicating a higher level of nitrogen starvation across genotypes. However, there was no difference in carbon content between all plants and all treatments (Figure 6b). Thus, nitrogen is the significant driver of the C/N ratio in Figure 6c, which shows all plants changing compositions significantly as nutrients are depleted. As mentioned in the introduction, nodulation is a costly process for the plant [2]. Since these nutrient stressed plants suppressed nodulation, we postulated that the plant reaches a point at which the resources required to mount the nodulation response are not available even though there is a nitrogen deficit that would otherwise promote nodulation. Indeed Voisin, *et al.* [30] showed a strong interaction between N and C metabolism in pea nodule number regulation and variation dependent on growth rates in the week preceding an inoculation. Also, nitrogen is not the only nutrient the “water only” plants are deficient in; phosphorus deficiencies have also been shown to affect the ability to nodulate [35], and these plants are likely low in potassium and some micronutrients as well.

The nutrient stress response observed when inoculating with water is at first glance puzzling: we reasoned that responder roots on plants inoculated with bacteria that could not fix nitrogen (Figure 4) should be as nutrient stressed as those in Figure 5, making the lack of nodule suppression unexpected in the roots responding to inoculation with Fix^−^ or Nod^−^ bacteria. Inoculation of *M. sativa* with Nod^−^*rhizobia* or even non-interacting Agrobacterium increased nodule development when co-inoculated with wild type bacteria [36] and it may be that the Fix^−^ or Nod^−^ inoculations “prime” the plant for future nodulation. While Caetano-Anollés and Bauer did not use a split root system with separate inoculations, our results could suggest a systemic effect, but we recognize this is speculation and further work is required. We note that many environmental variations, even variations in light intensity, can affect nodule number [30], and the experiments in Figure 4 and Figure 5 were performed in the greenhouse in different years, with likely small variations in other environmental inputs besides nutrients, as well as being inoculated with different species of *rhizobia*, which may contribute to the varied results.

## 3. Experimental Section 

### 3.1. Plant Materials and Growth Conditions

Seeds of *Medicago truncatula.*cv Jemalong A17 and AON defective mutants *rdn1-2* [14] and *sunn-4* [11] were acid scarified and imbibed as described in [20]. Seed was then vernalized in the dark at 4 °C for 2 days on Harrison Modified Farhaeus (HMF) media [37] covered with two half round Whatman filter papers (GE Healthcare, USA), followed by germination in the dark at room temperature for 1 day before being used in the experiments described.

### 3.2. Split Root Development and Inoculation 

Lateral root initiation was stimulated by removing the roots of 5-day-old seedlings at the root-shoot junction. The trimmed plants were placed onto HMF plates sandwiched between two half rounds of Whatman filter paper and split-root systems were established as described previously [27]. Plants were transferred to pots filled with washed, autoclaved perlite and placed in a greenhouse. They were watered daily with a 100-fold dilution of water-soluble 20:10:20 (N:P:K) Peat-Lite Special fertilizer (Scotts Company, OH, USA) for 5 days. After an additional 4 days of nutrient starvation induced by watering with water alone, the plants were used for split-root inoculation experiments with bacteria. The second root (Root B) was inoculated with *rhizobia* 0, 2, 3, 4, 6, 8, 10, 12, 15, or 20 days after the first root (Root A) inoculation and nodules on both roots were counted 21 days after the second root inoculation. Root systems that were not similar in length (within 20% of each other) at the 21-day time point were eliminated from consideration. For simultaneous inoculation (0 time point) one root at random was designated A and the other B.

### 3.3. Rhizobial Strains and Growth Conditions 

To demonstrate the timing of AON, both Root A and Root B were inoculated with *S. medicae* strain ABS7 [38]. To investigate the nature of a second nodule inhibitory signal, the tester root was inoculated with *Rm*1021 [32] or its derivatives *Rm*1312 [33], or SL44 [34] and the responder root with the wild type strain *Rm*1021; nodules on the responder root were counted after 21 days. Root systems that were unequal in size at the 21-day time point were eliminated from consideration.

All strains were grown in liquid TY media (5 g tryptone, 3 g yeast extract, 1.4 g CaCl_2_•2H_2_O, pH 6.5–7 and 15 g agar per liter) containing antibiotics at the following concentrations: tetracycline (15 μg/mL) for *S. medicae* and streptomycin (500 μg/mL) for *S. meliloti* strains. Before inoculation, the *rhizobia* were diluted to 0.2 OD 600 nm with sterile water and 6 mL of the bacterial solution was applied to each root compartment at root collar region (flood inoculation). Nodules were counted using an Olympus SZX12 Dissecting Stereo Microscope 21 days after the second root inoculation after gentle washing of the Perlite from the roots.

### 3.4. Nitrogen Fixation Measurements

The Q-Box NF1LP package (Qubit Systems Inc, Kingston, Canada) was used for measuring the rate of H_2_ production in order to estimate nitrogen fixation. Plants were prepared as described for the split root experiments with the same establishment and nutrient withdrawal regime and then inoculated with *S. medicae*. The flow-through H_2_ from N_2_-fixing tissues was measured on each plant at 10 days and 15 days post rhizobial inoculation in two independent experiments. The nodulated root systems were sealed with Qubitac^TM^ sealant to avoid air leakage so that only the shoots were exposed to room air. To generate maximum H_2_ evolution, roots were flushed with an Ar:O_2_ (80:20) gas combination at the rate of 250 mL/minute for 5 min, so that all electrons measured were utilized for H^+^ reduction. The measurement of H_2_ evolution as a means of determining nitrogenase activity used the LabPro interface in conjunction with Logger Pro software provided by Vernier (Portland, Oregon) according to the manufacturer’s instructions.

### 3.5. Determination of C and N

For each condition analyzed, three samples consisting each of pooled leaves from three plants (two trifoliates per plant) were dried, crushed, and prepared in tin capsules for C and N composition analysis by light stable isotope ratio mass spectrometry at the Duke Environmental Stable Isotope Laboratory [39]. Each sample weighed between 5.0 to 12.2 mg.

### 3.6. Statistical Analysis 

Statistical analyses were conducted to determine if the true means of the various responses (percent nodulation of the second root, nodule number, number of nitrogen fixing nodules, nodule fresh weight, hydrogen production, nodule number on responder root) differed among the levels of the various experimental factors of interest (days between inoculation of roots, dpi, and genotypes). Analysis of Variance (ANOVA) was used on the split root data (with Type I error rate set at 0.05) to determine if overall significant differences existed among the factor levels. If overall differences were found, pairwise comparison among the least squares means of the factor levels were performed using JMP-9 software (SAS Institute Inc., Cary, IL, USA). For all other analyses, the Student’s *t* test with the Bonferroni correction for multiple comparisons was used to determine the significance of differences from wild types. Calculation of error in mean percentages was done with a Taylor expansion of the variance.

## 4. Conclusions 

We show nodule regulation in *M. truncatula* in our system includes both an early response, related to nodule meristem development (the SUNN/RDN1 pathway) and a late response which does not require SUNN or RDN1 and is correlated with nutrient status, especially nitrate status. While the two signals are redundant in nodulating wild type plants, the response of *rdn1* and *sunn* mutants in the split root system allowed us to dissect out the timing of this second suppression event to coincide with the establishment of nodules actively fixing nitrogen. This timing would allow plants to continue to nodulate if the initial *rhizobia* are not providing nitrogen compounds for the plants or the nodules or roots are damaged and no longer fix an adequate supply.

Both nutrient stress and nitrate are implicated as regulating nodule number outside of the systemic CLE12/13-SUNN signaling pathway, and our experiments provide evidence for at least one additional pathway for nodule suppression that does not require SUNN or RDN1. Determining if the nitrate and/or nutrient stress pathways signal through common components or are different pathways will require additional experimentation with mutants in these other pathways. The recent isolation of mutants in *MtCRA2*, a leucine-rich repeat receptor kinase involved in nitrogen sensing and response, and the discovery of the *Mt*CEP1 peptide involved in nitrogen sensing should facilitate future studies to further understanding [40,41].

## References

[1] Oldroyd G., Downie A. (2008). Coordinating nodule morphogenesis with rhizobial infection in legumes. Annu. Rev. Plant Biol..

[2] Crawford N.M., Kahn M.L., Leustek T., Long S.R., Buchanan B.B., Gruissem W., Jones R.L. (2000). Nitrogen and sulfur. Biochemistry and Molecular Biology of Plants.

[3] Kosslak R.M., Bohlool B.B. (1984). Suppression of nodule development of one side of a split root system of soybeans caused by prior inoculation of the other side. Plant Physiol..

[4] Bhuvaneswari T.V., Bhagwat A.A., Bauer W. (1981). Transient susceptibility of root-cells in 4 common legumes to nodulation by rhizobia. Plant Physiol..

[5] Suzuki A., Hara H., Kinoue T., Abe M., Uchiumi T., Kucho K., Higashi S., Hirsch A.M., Arima S. (2008). Split-root study of autoregulation of nodulation in the model legume *Lotus japonicus*. J. Plant Res..

[6] Sargent L., Huang S.Z., Rolfe B.G., Djordjevic M.A. (1987). Split-root assays using *Trifolium subterraneum* show that rhizobium infection induces a systemic response that can inhibit nodulation of another invasive rhizobium strain. Appl. Environ. Microbiol..

[7] Van Brussel A.A.N., Tak T., Boot K.J.M., Kijne J.W. (2002). Autoregulation of root nodule formation: Signals of both symbiotic partners studied in a split-root system of *Vicia sativa* subsp. *Nigra*. Mol. Plant-Microbe Interact..

[8] Krusell L., Madsen L.H., Sato S., Aubert G., Genua A., Szczyglowski K., Duc G., Kaneko T., Tabata S., De Bruijn F.J. (2002). Shoot control of root development and nodulation is mediated by a receptor-like kinase. Nature.

[9] Nishimura R., Hayashi M., Wu G.J., Kouchi H., Imaizumu-Anraku H., Murakami Y., Kawasaki S., Akao S., Ohmori M., Nagasawa M., Pajuelo E., Sandal N., Stougaard J. (2002). HAR1 mediates systemic regulation of symbiotic organ development. Nature.

[10] Searle I.R., Men A.E., Laniya T., Buzas D., Iturbe-Ormaetxe I., Carroll B.J., Gresshoff P.M. (2003). Long-distance signaling in nodulation directed by a clavata1-like receptor kinase. Science.

[11] Schnabel E., Journet E.P., Carvalho-Niebel F., Duc G., Frugoli J. (2005). The *Medicago truncatula SUNN* gene encoding a *CLV1*-like leucine-rich repeat receptor kinase regulates both nodule number and root length. Plant Mol. Biol..

[12] Okamoto S., Shinohara H., Mori T., Matsubayashi Y., Kawaguchi M. (2013). Root-derived cle glycopeptides control nodulation by direct binding to HAR1 receptor kinase. Nat. Commun..

[13] Ferguson B.J., Indrasmunar A., Hayashi S., Lin M.H., Reid D.E., Gresshoff P.M. (2010). Molecular analysis of legume nodule development and autoregulation. J. Integr. Plant Biol..

[14] Schnabel E., Kassaw T., Smith L., Marsh J., Oldroyd G., Long S., Frugoli J. (2011). The *ROOT DETERMINED NODULATION1* gene regulates nodule number in roots of *Medicago truncatula* and defines a highly conserved, uncharacterized plant gene family. Plant Physiol..

[15] Ogawa-Ohnishi M., Matsushita W., Matsubayashi Y. (2013). Identification of three hydroxyproline *o*-arabinosyltransferases in *Arabidopsis thaliana*. Nat. Chem. Biol..

[16] Mortier V., Herder G., Whitford R., van de Velde W., Rombauts S., D’haeseleer K., Hosters M., Goormachtig S. (2010). CLE peptides control *Medicago truncatula* nodulation locally and systemically. Plant Physiol..

[17] Lim C.W., Lee Y.W., Hwang C.H. (2011). Soybean nodule-enhanced CLE peptides in roots act as signals in *Gm*NARK-mediated nodulation suppression. Plant Cell Physiol..

[18] Saur I.M.L., Oakes M., Djordjevic M.A., Imin N. (2011). Crosstalk between the nodulation signaling pathway and the autoregulation of nodulation in *Medicago truncatula*. New Phytol..

[19] Mortier V., de Wever E., Vuylsteke M., Holsters M., Goormachtig S. (2012). Nodule numbers are governed by interaction between CLE peptides and cytokinin signaling. Plant J..

[20] Schnabel E., Mukherjee A., Smith L., Kassaw T., Long S., Frugoli J. (2010). The *lss* supernodulation mutant of *Medicago truncatula* reduces expression of the *SUNN* gene. Plant Physiol..

[21] Schultze M., Kondorosi A. (1998). Regulation of symbiotic root nodule development. Annu. Rev. Genet..

[22] Carroll B.J., McNeil D.L., Gresshoff P.M. (1985). A super-nodulation and nitrate-tolerant symbiotic (nts) soybean mutant. Plant Physiol..

[23] Caba J.M., Recalde L., Ligero F. (1998). Nitrate-induced ethylene biosynthesis and the control of nodulation in alfalfa. Plant Cell Environ..

[24] Baker B., Zambryski P., Staskawicz B., Dinesh-Kumar S.P. (1997). Signaling in plant-microbe interactions. Science.

[25] Singleton P.W., van Kessel C. (1987). Effect of localized nitrogen availability to soybean half-root systems on photosynthate partitioning to roots and nodules. Plant Physiol..

[26] Laguerre G., Heulin-Gotty K., Brunel B., Klonowska A., Le Quere A., Tillard P., Prin Y., Cleyet-Marel J.C., Lepetit M. (2012). Local and systemic N signaling are involved in *Medicago truncatula* preference for the most efficient *Sinorhizobium* symbiotic partners. New Phytol..

[27] Kassaw T., Frugoli J. (2012). Simple and efficient methods to generate split roots and grafted plants useful for long-distance signaling studies in *Medicago truncatula* and other small plants. Plant Methods.

[28] Olsson J.E., Nakao P., Bohlool B.B., Gresshoff P.M. (1989). Lack of systemic supression of nodulation in split root systems of supernodulating soybean (*Glycine max* [l.] *merr.*) mutants. Plant Physiol..

[29] Hinson K. (1975). Nodulation responses from nitrogen applied to soybean half-root systems. Agron. J..

[30] Voisin A.S., Munier-Jolain N.G., Salon C. (2010). The nodulation process is tightly adjusted to plant growth. An analysis using environmentally and genetically induced variation of nodule number and biomass in pea. Plant Soil..

[31] Jeudy C., Ruffel S., Freixes S., Tillard P., Santoni A.L., Morel S., Journet E.P., Duc G., Gojon A., Lepetit M., Salon C. (2010). Adaptation of *Medicago truncatula* to nitrogen limitation is modulated via local and systemic nodule developmental responses. New Phytol..

[32] Meade H.M., Long S.R., Ruvkun G.B., Brown S.E., Ausubel F.M. (1982). Physical and genetic characterization of symbiotic and auxotrophic mutants of *Rhizobium meliloti* induced by transposon Tn5 mutagenesis. J. Bacteriol..

[33] Ruvkun G.B., Long S.R., Meade H.M., van den Bos R.C., Ausubel F.M. (1982). Isrm1: A *Rhizobium meliloti* insertion sequence that transposes preferentially into nitrogen fixation genes. J. Mol. Appl. Genet..

[34] Fisher R.F., Egelhoff T.T., Mulligan J.T., Long S.R. (1988). Specific binding of proteins from *Rhizobium meliloti* cell-free extracts containing NodD to DNA sequences upstream of inducible nodulation genes. Genes Dev..

[35] Tsvetkova G.E., Georgiev G.I. (2003). Effect of phosphorus nutrition on the nodulation, nitrogen fixation and nutrient-use efficiency of *Bradyrhizobium japonicum*_soybean (*Glycine max* l. *Merr.*) symbiosis. Bulg. J. Plant Physiol..

[36] Caetano-Anollés G., Bauer W.D. (1988). Enhanced nodule initiation on alfalfa by wild-type *Rhizobium meliloti* co-inoculated with Nod gene mutants and other bacteria. Planta.

[37] Huo X., Schnabel E., Hughes K., Frugoli J. (2006). RNAi phenotypes and the localization of a protein::GUS fusion imply a role for *Medicago truncatula PIN* genes in nodulation. J. Plant Growth Regul..

[38] Bekki A., Trichant J.C., Rigaud J. (1987). Nitrogen fixation (C_2_H_2_ reduction) by Medicago nodules and bacteroids under sodium chloride stress. Physiol. Plant..

[39] Duke Environmental Stable Isotope Laboratory. http://nicholas.duke.edu/devil/.

[40] Hualt E., Laffont C., Wen J., Mysore K.S., Ratet P., Duc G., Frugier F. (2014). Local and systemic regulation of plant root system architecture and symbiotic nodulation by a receptor-like kinase. PLOS Genet..

[41] Mohd-Radzman N.A., Binos S., Truong T.T., Iman N., Mariani M., Djordjevic M.A. (2015). Novel MtCEP1 peptides produced *in vivo* differentially regulate root development in Medicago truncatula. J. Exp. Bot..

